# The stretcher spontaneous neurodegenerative mutation models Charcot-Marie-Tooth disease type 4D

**DOI:** 10.12688/f1000research.2-46.v1

**Published:** 2013-02-13

**Authors:** David Chandler, Sash Lopaticki, Dexing Huang, Michael Hunter, Dora Angelicheva, Trevor Kilpatrick, Rosalind HM King, Luba Kalaydjieva, Grant Morahan

**Affiliations:** 1 Western Australian Institute for Medical Research and Centre for Diabetes Research, University of Western Australia, Perth, 6000, Australia; 2 Centre for Medical Research, University of Western Australia, Perth, 6000, Australia; 3 The Walter and Eliza Hall Institute of Medical Research, Victoria, 3065, Australia; 4 St Vincent's Institute of Medical Research, Victoria, 3010, Australia; 5 Howard Florey Institute, Victoria, 3010, Australia; 6 Department of Clinical Neurosciences, Institute of Neurology University College London, London, NW3 2PF, UK; 7 Australian Genome Research Facility, Perth, 6000, Australia

## Abstract

Mice affected by a spontaneous mutation which arose within our colony exhibited a neuromuscular phenotype involving tremor and characteristic stretching of the rear limbs. The mutant, named *stretcher*, was used to breed a backcross cohort for genetic mapping studies. The gene responsible for the mutant phenotype was mapped to a small region on mouse chromosome 15, with a LOD score above 20. Candidate genes within the region included the *Ndrg1* gene. Examination of this gene in the mutant mouse strain revealed that exons 10 to 14 had been deleted. Mutations in the human orthologue are known to result in Charcot-Marie-Tooth disease type 4D (CMT4D) a severe early-onset disorder involving Schwann cell dysfunction and extensive demyelination. The *stretcher *mutant mouse is more severely affected than mice in which the *Ndrg1* gene had been knocked out by homologous recombination. Our results demonstrate that the *Ndrg1* ^str^ mutation provides a new model for CMT4D, and demonstrate that exons 10 to 14 of *Ndrg1* encode amino acids crucial to the appropriate function of Ndrg1 in the central nervous system.

## Introduction

Over 60 spontaneous mouse mutations that exhibit neurological disorders including movement abnormalities or epilepsy conditions are listed in the Mouse Genome Informatics database. Most of these mutations have been defined at the molecular level. Identifying the genes affected has provided insights into the molecular basis of neurological functions; some examples are reviewed in ^1, 2^. The availability of animal models of disease aids in understanding its molecular basis and is valuable in the search for new treatments. Nevertheless, many neurological diseases of humans still lack satisfactory animal models.

Previously we had mapped a locus, *Idd11*, which conferred susceptibility to type 1 diabetes in the NOD/LtJ mouse strain ^3^. During the production of congenic mice bearing the C57BL/6J (B6) resistance allele of *Idd11* ^3, 4^ on the NOD background in our laboratory, a spontaneous mutation arose. These mutant mice exhibited a neurological defect. This paper describes the phenotypic characterization of these mutant mice, as well as mapping, identification and characterization of the mutant gene.

## Materials and methods

### Mice

Mouse work was performed with ethics approval from the Royal Melbourne Hospital Animal Ethics Committee and from the Animal Ethics Committee of The University of Western Australia. All procedures conformed to the Guidelines for the Care and Use of Experimental Animals described by the National Health and Medical Research Council of Australia. BALB/c, C57BL/6J (B6), DBA/2 and NOD/LtJ (NOD) mice were obtained from either the specific-pathogen free colonies of The Walter and Eliza Hall Institute of Medical Research or from the Animal Resources Centre (Murdoch, Western Australia). NOD. *Slc9a1 ^b^* congenic mice ^4^ were maintained in conventional M1 "shoe box" mouse cage (335mm Long × 160mm Wide × 130mm High).

Each cage comprised of 1 male and 1 female with litters being weaned from the box at 3 weeks of age. All animals were provided with food and water *ad libitum*, aspen wood bedding and an environment enrichment consisting of tissue paper for nesting. All animals were cared for by specialist trained staff with experience in clinical observations of ill health, and behaviour irregularities. A vet was on site to provide an opinion to any observations and instigate necropsy if required. Animals that exhibited ill health were euthanased in pre-charged carbon dioxide chambers. The mice displaying the neurological defect, named *stretcher (str)*, were intercrossed with BALB/c mice obtained from The Walter and Eliza Hall Institute of Medical Research. A congenic strain, BALB/c. *str* was developed after 10 generations of backcrossing to BALB/c, selecting for retention of NOD-derived alleles at markers on chromosome 15. To map the *str* mutation, we chose to mate NOD. *Slc9a1 ^b^* mutant mice to a third strain, DBA/2 (D2). This was done because the NOD. *Slc9a1 ^b^* mice already had an introduced B6 chromosome region which could potentially complicate mapping.

### Genotyping and gene mapping

Conventional microsatellite genotyping was performed using MIT markers ^5^ under standard conditions as previously described ^3^. Novel markers were also developed as follows and are listed in Table 1. cDNA sequences of genes previously mapped to the region were BLASTed against GENBANK DNA databases to retrieve genomic sequences. Genomic sequences were also retrieved from the mouse genome sequence ^6^ as reported in the NCBI 37 July 2007 assembly ( UCSC Genome Browser). Simple sequence length repeats were selected and primers were designed using the Primer3 program ^7^. Primer sequences are listed in Table 1. These were used to amplify the relevant alleles from NOD and DBA/2 DNA. LOD scores and significance thresholds were calculated as described by Lander and Kruglyak (1995) ^8^.

**Table 1.** Primers used to amplify novel markers. Simple sequence length repeats were found from inspection of relevant genomic sequences. The location of the nearest known gene, the genomic position (in Mb from the UCSC July 2007 freeze) of the repeat; the primers used to amplify it; the annealing temperature used (Tm), and the sizes of alleles from B6, DBA/2 and NOD mice, are indicated.

Marker	Gene	Mb	Left primer (5´-3´)	Right primer (5´-3´)	Tm	B6	DBA/2	NOD
*D15Mor1*	*Kcnq3*	66.1	ATGTGTGCTGCTTTGAGCTG	TGCTATGTATCCACACAGCAAA	60	235	235	180
*D15Mor2*	*Tgn*	66.5	CTGCCATGGCTTCATTTTCT	GCAAATGCCAGGGTTCTGTA	60	230	220	230
*D15Mor3*	*B8C052212*	66.6	CCATACCCCAGAAAGAAAAG	TCCTTTAACATGATGGGAGA	56	128	128	128
*D15Mor4*	*Ndrg1*	66.8	AGGTCAGACAGGGTCAGCTAAG	CACATCCTCTCCCACTGAGG	60	155	155	150
*D15Mor5*	*Siat4*	66.9	ATGTCTGCTGAGTGCTGAGG	GATGCCACCCTCCTACACAT	59	194	194	235
*D15Mor6*	*Etoile*	68.5	CCAGGATTTCTTTGGTCTTCTTT	CATGTACCAGGTGCCATGAA	60	193	193	188

### RNA isolation, cDNA synthesis and sequencing

Whole kidneys from wild-type BALB/c mice or mutant mice euthanised by exposure to carbon dioxide gas were homogenised in 500µl of Triazol (Gibco) reagent and RNA was extracted according to the manufacturer’s instructions. For cDNA synthesis, 2µg of RNA was reverse transcribed using 1 × reverse transcription buffer (Promega), 1U of RNase inhibitor (Invitrogen), 2mM of dNTPs, 50ng/µl of random hexamers (Promega), and 8U of MMLV-reverse transciptase (Promega) in a total volume of 20µl. Reaction mixes were incubated at 42°C for 60 minutes and the reaction stopped by heat inactivation at 95°C for 10 minutes. The cDNA was used as a template for the amplification of a PCR product spanning exon 6 to 15 of the *Ndrg1* gene. The reaction consisted of 1x PCR buffer, 2.5mM MgCl2, 5mM dNTPs, 1.5U Taq polymerase (Kappa), 20ng of each primer (5' GAGGACATGCAGGAGATCAC 3' and 5' CAGAGGCTGTGCGGGACC 3') and water in a total volume of 50µl. PCR cycling conditions consisted of initial denaturation at 95°C followed by 40 cycles of denaturation at 95°C for 30 seconds, annealing at 55°C for 30 seconds and extension at 72°C for 45 seconds with a final extension step at 72°C for 7 minutes. The products were cleaned with PCR purification columns (Qiagen) and sequenced using BigDye Terminator chemistry (Life Technologies).

### Northern blotting

For northern blotting, 5µg of RNA was electrophoresed on a 1.2% agarose/formamide gel for 2 hours in MOPS buffer. The RNA was transferred to a nitrocellulose membrane via capillary-wick blotting in SSC buffer (Sigma-Aldrich) for 3 hours and the membrane was dried in an oven set at 80ºC for ten minutes. The RNA was fixed onto the membrane by a 4 minute exposure to UV light (312nm) in a UV cabinet. A DNA probe was constructed from a 233bp PCR product spanning exons 2 to 4 of *Ndrg1* amplified from mouse kidney cDNA using primers 5' GACCTCGCTGAGGTGAAGCC 3' and 5' GTGATCTCCTGCATGTCCTC 3'. The PCR product was labelled with ^32^P-CTP using a Random Primed Labelling kit (Roche) according to the manufacturer’s instructions. The membrane was incubated in 3ml of Ultrahyb® hybridization solution (Ambion) for 30 minutes at 42ºC and replaced with 5ml of fresh solution containing the denatured labelled probe (activity of 6.0 × 10 ^5^ cpm/ml). Hybridization was carried out with rotation at 42ºC for 24 hours. The membrane was then washed twice in 2x SCC, 0.1% SDS buffer pre-warmed to 42ºC for 10 minutes and twice with 0.1xSCC, 0.1% SDS buffer for 15 minutes. The membrane was wrapped in cling film and exposed to Medical X-Ray film for 16 hours at –80ºC. The film was developed on an AGFA CP100 processor.

### Western blotting

For isolation of total protein, sciatic nerves were dissected and homogenised in RIPA buffer (1% Nonidet P-40, 0.1%SDS, 0.5% deoxycholate (Sigma-Aldrich), 150mM NaCl, 50mM Tris pH 8.0, 10µg/ml aprotinin, 1mM PMSF, 1mM benzamidine (Sigma-Aldrich), 0.1mM Na _3_VO _4_) and centrifuged at 13,000rpm for 20 minutes at 4ºC. The supernatant was transferred to a new tube and quantitated. 10µg of protein was loaded into single wells of a 12% SDS-PAGE stacking gel (Invitrogen) and electrophoresed at 125V for 30 minutes, and 200V for approximately 1 hr. Proteins were transferred to PVDF membranes (Invitrogen) by western blotting at 30V overnight at 4°C. The membranes were probed first with an affinity-purified polyclonal rabbit antibody raised against the full-length NDRG1 protein (A gift from K. Kokame and T. Miyata). After exposure and subsequent stripping, the membrane was then re-probed with a goat polyclonal IgG directed against the N-terminus of the human NDRG1 protein (Santa Cruz Biotech). Immuno-labelled protein bands were visualised using the ECL+ Chemiluminescence kit (Amersham Biosciences) and exposure to Hyperfilm™ ECL Chemiluminescence film (Amersham Biosciences).

### DNA sequencing

Primers were derived from the *Ndrg1* genomic sequence and used to amplify DNA from B6, NOD/LtJ, and BALB/c. *str* mice. Sequencing was performed using the Big Dye terminator kit (Life Technologies) followed by capillary electrophoresis on a 3730 DNA analyser (Life Technologies).

### Bioinformatics and model construction

Hypothetical protein models were constructed from the *Ndrg1 ^str^* cDNA sequence using The HMMSTR/Rosetta Server (available at http://www.bioinfo.rpi.edu/bystrc/hmmstr/server.php) This software implements the HMMSTR (a hidden Markov model for local and secondary structure prediction) and Rosetta (a Monte Carlo Fragment Insertion protein folding program) programs to predict the structure of proteins ^9^. Wild-type and mutant protein sequences were analysed at http://www.predictprotein.org/ to determine whether esterase classification was retained.

## Results

### Phenotype of the mutant *stretcher* mice

**Figure 1.**
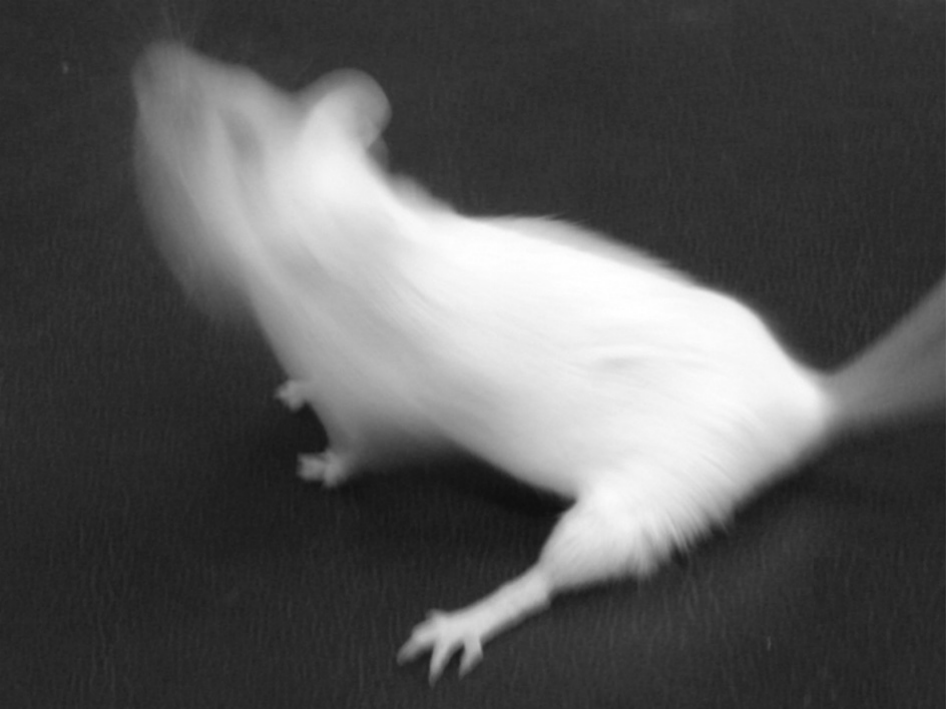
*Stretcher* mutant mouse phenotype. The *stretcher* mutant is characterized by the stretching and “freezing” of the hind limbs, as illustrated in this photograph. The trait is most apparent when the mice are challenged with some behavioural intervention (e.g. handling for clinical examination).

The spontaneous mutation was observed in our NOD. *Slc9a1 ^b^* congenic mouse strain ^4^ (referred to as NOD. *Idd11*B in that paper). The mice showed a characteristic stretching of the rear limbs, especially when they were handled for examination ( Figure 1). This feature inspired the mutant strain to be named *stretcher* ( *str*). The characteristic stretching was accompanied by tremor. Mice also clasped their hind limbs when suspended. The phenotype was most noticeable after 5 weeks of age and progressively worsened, so that after 15 weeks the mice became weak and showed severe tremor of the hind limbs.

Because the strain in which the mutation arose develops type 1 diabetes ^4^, there was a danger of losing the mutant stocks, so we introgressed the *str* mutation onto the nondiabetic strain, BALB/c. In general, though they are fertile, the male *str* mice have difficulty in mating. Therefore, the BALB/c. *str* strain was derived by 10 generations of backcrossing females to BALB/c males (selecting for linked markers that were developed as described below). This strain was maintained by sib mating, taking care to set up brother-sister pairs as soon as they reached breeding age.

### Mapping of the *str* gene

**Figure 2.**
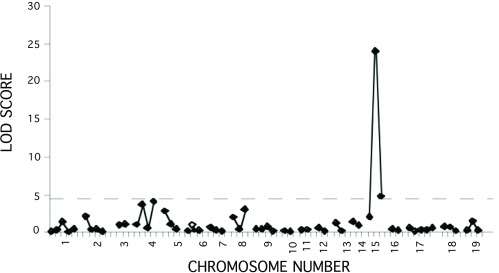
Genome scan to map the *str* mutation. Affected F2 progeny (n=49) of (NOD. *str* x DBA/2) F1 parents were typed with microsatellite markers with an average spacing of 20cM over the 19 autosomes. LOD scores were calculated and the dashed line shows the threshold for significance for an F2 genome-wide scan (Lander & Kruglyak, 1995) ^8^.

**Figure 3.**
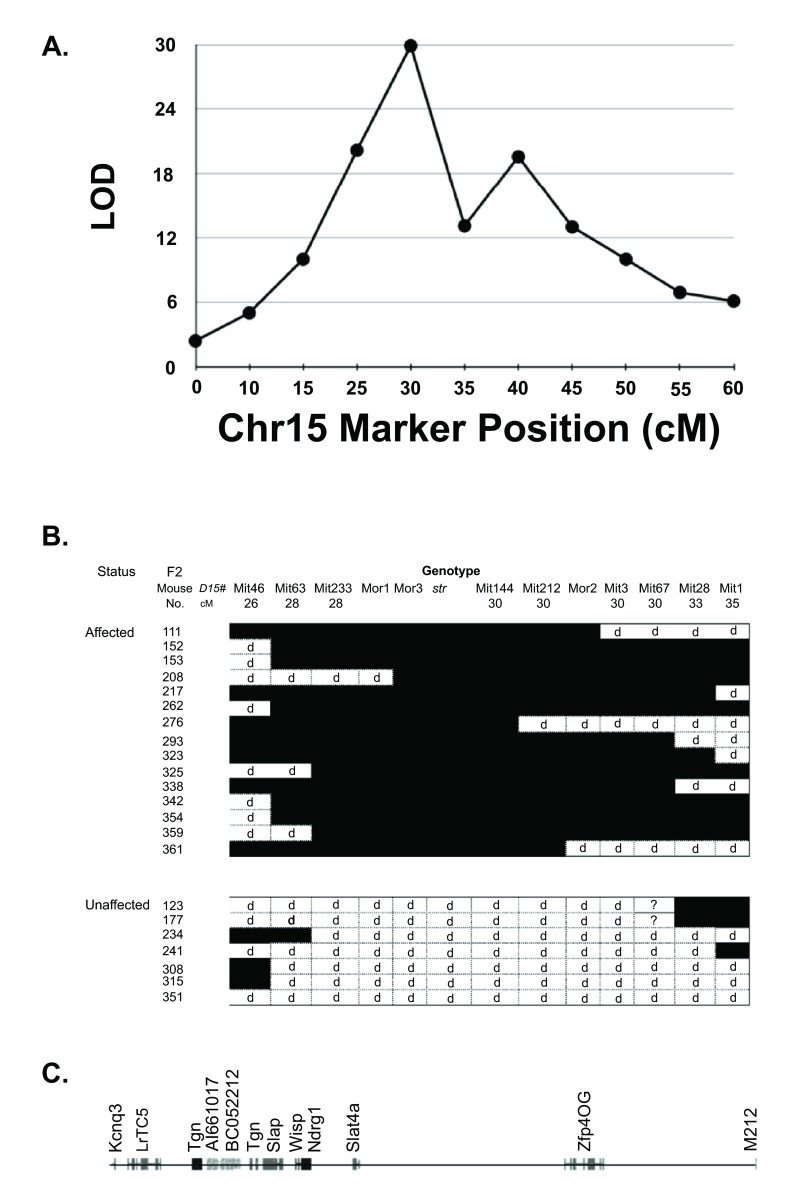
Fine mapping of *str* mutation. **A**. Affected F2 mice were typed with markers on chromosome 15 and LOD scores calculated as in Figure 1. **B**. Affected and unaffected mice which had recombinations within 5 cM of the peak of linkage were genotyped with additional markers. “ *Mit*” denotes *D15Mit* markers, with their given positions in cM; “ *Mor*” denotes novel *D15Mor* markers developed here (see also Table 1). Filled squares = homozygous for allele derived from the NOD. *str* strain; d = at least one copy of DBA/2 allele. **C**. Genomic map of chromosome 15 between the flanking markers *D15Mor1* (which is in an intron of *Kcnq3*) and *D15Mit212*, from 66.1Mb to 68.5 Mb of the UCSC October 2007 Assembly.

At the same time as the congenic mice were being produced, affected NOD. *Slc9a1 ^b^* mice were also mated with DBA/2 mice in order to map the *str* locus. The F1 offspring were unaffected, so F2 progeny were produced and observed for the *stretcher* phenotype. DNA samples from 58 affected F2 mice and 269 unaffected mice were genotyped with markers distributed across the genome. Linkage was observed to markers only on chromosome 15 ( Figure 2) with a single-point LOD score = 23.9 at *D15Mit63*. High resolution genotyping was then performed on both affected and unaffected F2 mice. In this way, it was possible to map the *str* locus to an interval of approximately 2cM between the markers *D15Mit233* and *D15Mit144* ( Figure 3B). We developed simple sequence length repeat polymorphic markers associated with a number of genes that mapped to the general area, including *Kcnq3*, *Siat4a* and *Etoile* ( Table 1). By testing these markers on the panel of F2 mice carrying recombinations between the flanking markers, we excluded *Kcnq3* and *Etoile* as candidates for *str*, since these mapped either centromeric or telomeric of the critical region, respectively. The *D15Mor1* marker defined the new centromeric boundary of the region in which the *str* locus was mapped. The markers *D15Mor3* and – *4*, defining the *Wisp* and *Siat4a* genes respectively, were located within this interval.

### Sequencing the *Ndrg1* gene from *str* mice

Although the critical region covers 3Mb, this interval is relatively gene-poor with only 11 known gene transcripts ( Figure 3C). However, several of these genes could be considered candidates for the *str* mutation. Of these, *Ndrg1* was considered as an especially good candidate since mutations in the human orthologue have been shown to be the cause of a demyelinating peripheral neuropathy, Charcot-Marie-Tooth disease type 4D ^10^. This disorder is characterized clinically by distal muscle wasting and atrophy, tendon areflexia, and sensory loss, with onset before ten years of age. Therefore, DNA from *str* and wild-type NOD mice was amplified using primers designed to amplify *Ndrg1* exons from the genomic sequence.

**Figure 4.**
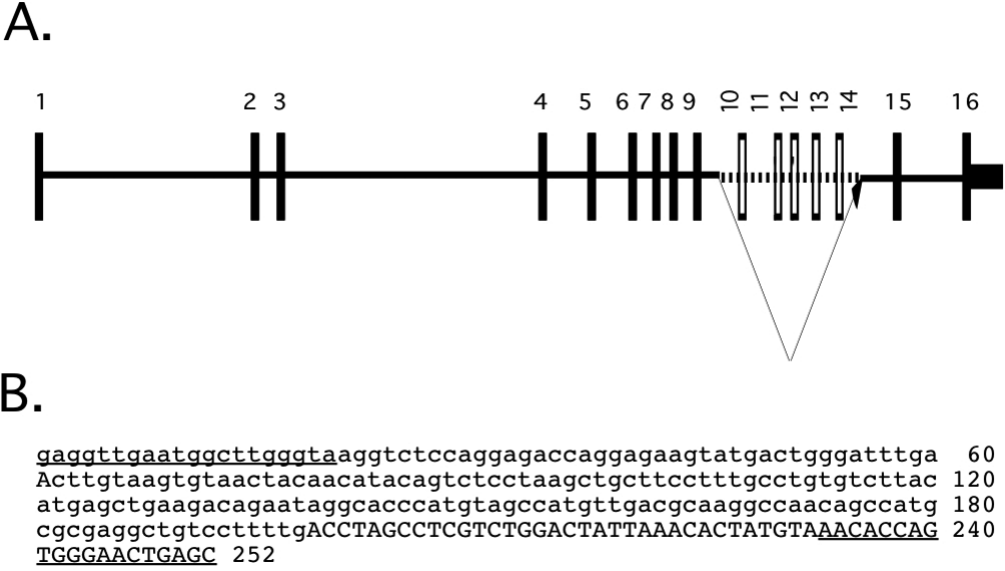
Sequence of *Ndrg1* allele from stretcher mice. **A**. Genomic organization of *Ndrg1* gene. Exons are represented by filled boxes. The extent of deletion between introns 9 and 14 is indicated; the deleted sequence is indicated by the dotted line and empty boxes. Sequence is shown reversed in comparison to chromosomal orientation. **B**. Sequence flanking the deletion point. Lower case: sequence from intron 9; upper case: intron 14 sequence; underline: sites for primers to amplify deletion allele.

Sequences of these amplicons were compared to the available genomic sequences but no polymorphisms which would result in amino acid substitutions were identified. During the course of this work, we were unable to amplify exons 10, 11, 12, 13 or 14 from the *str* mice. We reasoned the most likely explanation for this finding was that these exons had been deleted. A number of primers flanking exons 9 and 15 were designed and used in various combinations to test this hypothesis. Eventually, we were able to confirm that these exons had in fact been deleted, and to define the exact points between which the deletion had occurred. As shown in Figure 4, over 5kb of DNA encompassing exons 10 to 14 had been deleted. The deletion breakpoints are precise, with no addition of nontemplated nucleotides.

### Expression of mutant *Ndrg1* transcripts and protein

**Figure 5.**
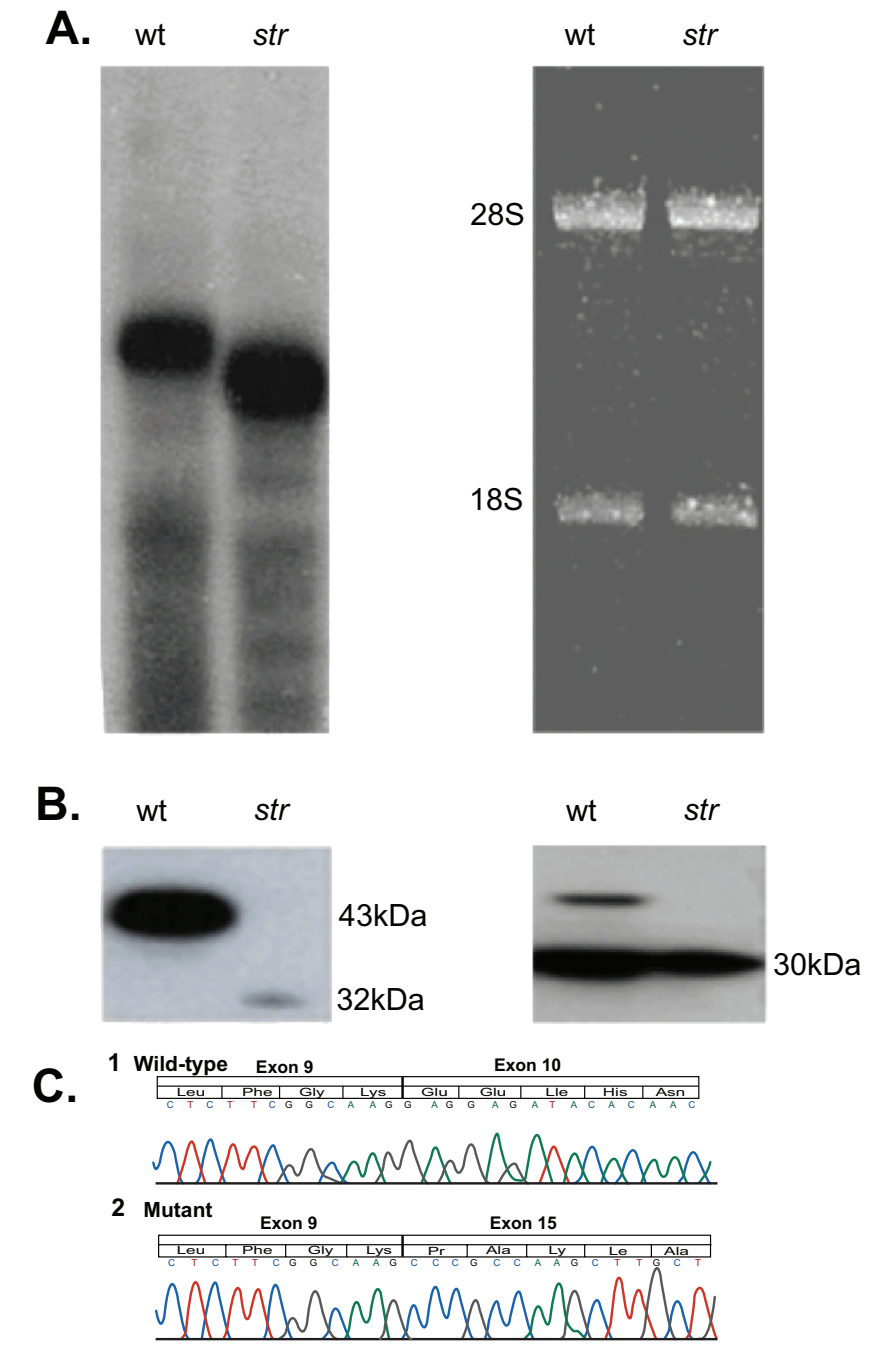
Analysis of *Ndrg1 ^str^* transcripts and protein product. **A**. A northern blot of RNA from kidneys of wild-type and *str* mice. A 233bp probe spanning exons 2–4 detected in the mutant mouse anRNA species shorter than that seen in the wild-type *Ndrg1* RNA ( *left panel*). Ethidium bromide-staining of the agarose gel prior to northern transfer showed equal amounts of RNA were loaded ( *right panel*). **B**. Western blot of sciatic nerve lysates prepared from wild-type and *str* mutant mice, probed with antibodies raised against the full-length Ndrg1 protein ( *left*) or GAPDH ( *right*). The full length (43 kDa) Ndrg1 protein was absent from the lysate of the *str* mice but an immunoreactive truncated (32 kDa) protein was present in a lower amount; this size is approximately that predicted for the Ndrg1 ^str^ mutant protein. **C**. Chromatograms and translated protein sequences of Ndrg1 cDNA prepared from kidney tissue from 1) wildtype BALB/C and 2) the mutant mouse. The deletion results in the skipping of exons 10–14. Exon 15 is spliced in-frame with exon 9.

The northern blot analysis revealed a shorter mRNA band, present in the *str* animals at levels similar to the normal product found in WT littermates ( Figure 5A). The sequence of Ndrg1 *^str^* cDNA confirmed that transcripts from the mutant allele were processed with in-frame splicing directly from exons 9 to 15 ( Figure 5C). A western blot analysis of protein extracted from sciatic nerve revealed a faint band at ~32 kDa, corresponding to the expected molecular mass of the mutant protein missing the 99 amino acids encoded by the deleted exons ( Figure 5B). Bioinformatic analysis of the abnormal protein showed it could remain classified as a member of the esterases/lipases superfamily. The one letter amino acid codes for both the ndrg1 WT and mutant proteins are displayed below:

### Ndrg1 WT

msrelhdvdlaevkplvekgesitgllqefdvqeqdietlhgslhvtlcgtpkgnrpviltyhdigmnhktcynplfnsedmqeitqhfavchvdapgqqdgapsfpvgymypsmdqlaemlpgvlhqfglksvigmgtgagayiltrfalnnpemveglvlmnvnpcaegwmdwaaskisgwtqalpdmvvshlfgkeeihnnvevvhtyrqhilndmnpsnlhlfisaynsrrdleierpmpgthtvtlqcpallvvgdnspavdavvecnskldptkttllkmadcgglpqisqpaklaeafkyfvqgmgympsasmtrlmrsrtasgssvtslegtrsrshtsegprsrshtsegsrsrshtsedarlnitpnsgatgnnagpksmevsc.

### Ndrg1 mutant

msrelhdvdlaevkplvekgesitgllqefdvqeqdietlhgslhvtlcgtpkgnrpviltyhdigmnhktcynplfnsedmqeitqhfavchvdapgqqdgapsfpvgymypsmdqlaemlpgvlhqfglksvigmgtgagayiltrfalnnpemveglvlmnvnpcaegwmdwaaskisgwtqalpdmvvshlfgkpaklaeafkyfvqgmgympsasmtrlmrsrtasgssvtslegtrsrshtsegprsrshtsegsrsrshtsedarlnitpnsgatgnnagpksmevsc.

Further biochemical and structural characterization of the effect of the stretcher mutation is described elsewhere ^11^.

### Hypothetical model of truncated Ndrg1

**Figure 6.**
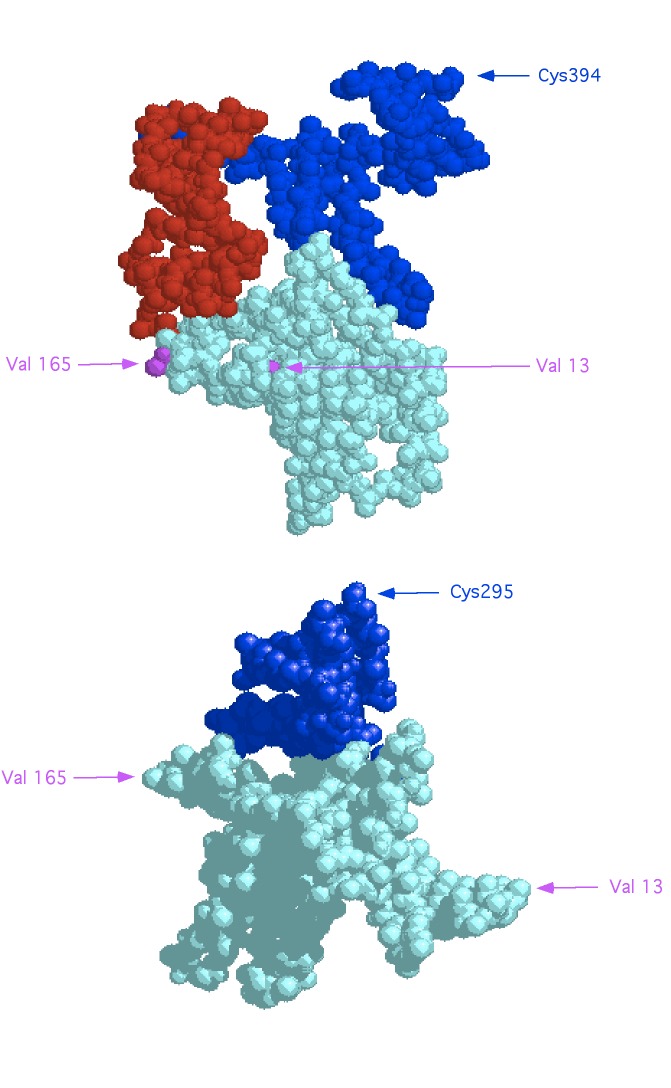
Hypothetical structure of wild-type Ndrg1 (upper) and the Ndrg1 *str* mutant (lower) proteins. Certain residues are indicated for reference. Cyan, amino acids prior to #199; red, residues encoded by exons deleted in the Ndrg1 *str* mutant; blue, residues 298-end of wild-type Ndrg1.

Splice sites for exons 9 and 15 were unaffected by the deletion, and sequencing of the transcripts from the truncated gene showed they could be spliced correctly but would encode a smaller protein product than would the wild-type gene. Hypothetical structures for the normal and mutant proteins were generated using the HMMSTR/Rosetta Server ^9^. These models are presented in Figure 6. The predicted structure of the truncated protein has an overall similarity to the wild-type, but also contains conformational changes in compensation for the deleted sequences. The major changes to the first third of the molecule may explain the functional deficit of the mutant Ndrg1 molecule in the *str* mice. In view of the low amounts detectable by western blot, the mutant protein is likely to be unstable.

## Discussion

Here we report the identification and characterization of the spontaneous mutant *stretcher* mouse, a new model of Charcot-Marie-Tooth 4D disease, with a spontaneous deletion of exons 10–14 of the *Ndrg1* gene. We showed that the *Ndrg1 ^str^* mutation results in low levels of expression of a truncated protein which, compared to the normal protein, is missing 99 amino acids (ie #199 to 297 of the wild-type sequence).

The absent Ndrg1 fragment is due to the deletion in the *Ndrg1 ^str^* allele. The protein fragment encoded by the deleted exons does not show homology to any particular conserved domain family. The functional importance of the missing domain is highlighted by both the *str* mutation and the human splicing mutation, 2290787G>A which skipped exon 9 ^12^. The reading frame was preserved in both mutations, yet the phenotype in each case was severe peripheral neuropathy. The low detectable levels of aberrant protein suggest that it is unstable, leading to the neurological phenotype observed only in homozygote mutant mice.

The *stretcher* mutation has been characterized by histology ^11^ and is more severely affected in both molecular and behavioural phenotypes than was reported for the *Ndrg1-/-* mouse ^13^. Though the comparisons should be made on the same genetic background, the milder phenotype of the *Ndrg1-/-* mouse is probably due to the knockout strategy which resulted in excision of the promoter and exon 1, but left intact the initiation codon in exon 2 as well as the rest of the coding region. It seems that these mice are able to produce sufficient amounts of full-length protein to avoid the more extreme phenotype displayed by the *stretcher* mutant mice, and only display the reported milder phenotype of muscle weakness.

We conclude the *Ndrg1 ^str^* mutant mouse will be a useful resource for investigating the role of *Ndrg1* in maintaining the myelin sheath, and for modelling the human disorder, Charcot-Marie-Tooth disease 4D.
