# Supplementary material for: The stretcher spontaneous neurodegenerative mutation models Charcot-Marie-Tooth disease type 4D
Source: F1000Res. 2013 Feb 13;2:46. [Version 1] doi: 10.12688/f1000research.2-46.v1 (PMC3976107; doi:10.12688/f1000research.2-46.v1)
Supplement: Peer review history

# Peer review: Referee response for version 1

**Published:** 2013-05-20

**Authors:** Angelo Schenone

**Affiliations:** 1 Department of Neurology, University of Genoa, Liguria, Italy

The title and abstract are appropriate for the content of the article and the abstract does represent a good summary of the work. Design, methods and analysis are nicely explained and indeed appropriate for the topic of the study. Results are definitely important to the research in the field of hereditary neuropathies, as they give insights on the role of the NDRG1 gene in the development of a rare type of CMT (CMT4D). Having good animal models for the different types of CMT and a detailed description of their phenotype (given in the paper published in Neurobiology of Disease- PMID21303696) is pivotal to unravel disease pathomechanisms and program future therapies. The discussion and conclusions are justified. However, it would have been very helpful to the readers reporting the presence of a canine model of CMT4D which carries a small deletion within the NDRG1  gene. Canine CMT is relatively frequent and, to the best of my knowledge, this is the only model in which a genetic change has been reported.

I have read this submission. I believe that I have an appropriate level of expertise to confirm that it is of an acceptable scientific standard.

# Peer review: Referee response for version 1

**Published:** 2013-05-07

**Authors:** Fransesc Palau

**Affiliations:** 1 Rare and Genetic Diseases Program , Centro de Investigación Príncipe Felipe (CIPF), Valencia, Spain

This paper provides new knowledge on the role of the *Ndrg1* gene in the biology of peripheral nerve, and more specifically in the pathophysiology of Charcot-Marie-Tooth disease type 4D (CMT4D) caused by mutations in *Ndrg1*. Most importantly, the stretcher mouse ( *Ndrg1* ^str^) provides a very useful tool to investigate in depth the myelin biology and defects associated to NDRG1 protein. 

From the experimental aspects, the authors show a nice and classical genetic approach to isolate and characterize the gene in a spontaneous mutant mouse, which include phenotyping, gene mapping, gene expression and molecular genetics to define the mutation. Further histological phenotypes have been reported elsewhere, as indicated by the authors ( Ref. 11- King *et al*.). It is noteworthy, the finding of a more severe phenotype in the stretcher mouse than in the *Ndrg1-/-,* which suggests the protein domain encoded by exons 10-14 are very important in protein function (a biological aspect that deserves more attention in future investigations). In this way, the paper remains a description of an animal model of CMT4D, and I want to state the relevance to move forward to the functional role of NDRG1 in the physiology of the Schwann cell and Schwann cell-axon interaction.

I have read this submission. I believe that I have an appropriate level of expertise to confirm that it is of an acceptable scientific standard.

# Peer review: Referee response for version 1

**Published:** 2013-04-17

**Authors:** Rhona Mirsky

**Affiliations:** 1 Department of Cell and Developmental Biology, University College London, London, UK

The title and abstract are appropriate for the content of the article. The article is clearly written, and the design, methods and analysis are appropriate.  The article shows that the mutation is due to deletion of exons 10-14 of the *Ndrg1* gene, resulting in an unstable smaller protein, obtained by translation of exons 1-9 and exons 15 and 16 in frame. The functional deficits in the hind limbs appear clearly at about 5 weeks of age – this represents a relatively early onset which can be compared to the early onset seen in human recessive Charcot-Marie-Tooth type 4D which shows an early onset. The model appears to model the human disease phenotype more faithfully than the previously reported *Ndrg1* knockout mouse and therefore represents an additional tool for research.

I have read this submission. I believe that I have an appropriate level of expertise to confirm that it is of an acceptable scientific standard.
